# Indicators of male fertility potential in adult patients with beta-thalassemia major: a comparative study between patients undergone allogeneic stem cell transplantation and transfusion-dependent patients

**DOI:** 10.1186/s40738-020-00071-6

**Published:** 2020-03-07

**Authors:** Tahereh Rostami, Mohammad Amir Mohammadifard, Shahla Ansari, Azadeh Kiumarsi, Nasrollah Maleki, Amir Kasaeian, Fatemeh Aghamahdi, Soroush Rad, Ardeshir Ghavamzadeh

**Affiliations:** 1 Hematology, Oncology and Stem Cell Transplantation Research Center, Shariati Hospital, Tehran University of Medical Sciences, Tehran, Iran; 2 Department of Hematology-Oncology, Ali Asghar Children’s Hospital, Iran University of Medical Sciences, Tehran, Iran; 3 Non-communicable Diseases Research Center, Alborz University of Medical Sciences, Karaj, Iran

**Keywords:** Thalassemia, HSCT, Fertility, Gonadal failure, Spermatogenesis, Tanner stage

## Abstract

**Background:**

Allogeneic hematopoietic stem cell transplantation (HSCT) remains the only potentially curative treatment for thalassemia major (TM). Infertility and its indicators have been assessed in transfusion dependent TM men, but in this study, we sought to compare the fertility indicators of TM patients after HSCT with those in patients treated conventionally. The possible influential factors on reproductive capacity in TM patients undergone allogeneic HSCT were also evaluated.

**Patients and methods:**

In this cross-sectional study, we compared the gonadal hormones level, testicular volume, Tanner stage and sperm analysis in transfusion-dependent thalassemia major (TDTM) patients who survived matched sibling HSCT (*n* = 43) with patients conventionally treated by transfusion and iron chelation (*n* = 52).

**Results:**

The patients’ age range was between 16 to 41 years. Tanner stage 4–5 was seen in 39 patients (41%). The prevalence of hypogonadism in our patients was 32.63% but its frequency was not significantly different between the two groups (*p* = 0.35). Azospermia, oligospermia, astenospermia, teratospermia and even having dry and low volume ejaculate were all significantly more frequent in the post-transplant patients compared to TDTM group. In the post-HSCT group, neither patients’ age at transplantation nor the conditioning regimen used in their transplant process did significantly affect their hormonal status and sperm parameters. Chronic graft versus host disease (GVHD) occurred in 14 (40%) patients. No significant difference was observed between the grade of chronic GVHD and hypogonadism (*P* = 0.853).

**Conclusions:**

Thalassemia patients undergone allogeneic HSCT have lower fertility potential, mainly in sperm parameters compared with patients treated with blood transfusion and chelation. This information is important for thalassemic patients considering HSCT.

## Introduction

Beta-thalassemia is a hereditary hemolytic anemia owing to marked reduced or absent beta globin chain synthesis [1]. The condition is primarily managed by regular blood transfusion and chelation. With implementation of remarkably improved medical care, patients with transfusion-dependent thalassemia major (TDTM) have reached the age of reproduction [2]. Furthermore, allogeneic hematopoietic stem cell transplantation (HSCT) as the only curative treatment for this hemoglobinopathy, has offered patients a near normal life span and the long-term survivors wish to have an unrestricted quality of life, including the experience of parenthood.

Normal reproduction requires germ cells and an intact hypothalamic-pituitary endocrine axis. However, the likelihood of successful fertility in thalassemia patients could be negatively influenced by different factors including iron overload and tissue hypoxia due to chronic anemia in conventionally treated patients. Iron deposits in the gonads, pituitary gland or both leads to hypogonadism and in TDTM patients, hypogonadotropic hypogonadism due to iron deposition in the pituitary gonadotrope cells is more frequently found. On the other hand, pretransplant myeloablative conditioning regimen (total body irradiation, busulfan, and alkylating agents) and post-transplant complications such as graft versus host disease (GvHD) and infections impact fertility potential in HSCT survivors [3–8]. The age of the patient at transplantation may play an additional role. Some researchers have reported a higher probability of residual fertility when the transplant is performed at a younger age [9].

Evaluation of fertility potentials after allogeneic HSCT is expounding, particularly for patients who could not access any strategy for fertility preservation prior to transplantation [10]. Alongside, specific protocols for managing fertility issues in male patients with TM undergoing allogeneic HSCT are exceedingly required.

To address these requisites, we sought to investigate the fertility indicators of TM patients after HSCT and compare the results with those in patients treated conventionally. As secondary objectives, we evaluated factors that might influence fertility in the two groups of patients, such as comorbidities associated with iron overload, different types of chelation therapies used in conventionally treated patients, conditioning regimen for transplanted patients and the age at HSCT.

## Materials and methods

### Study design and participants

This is a cross-sectional comparative study on the fertility indicators in TDTM patients who were recruited from Ali asghar hospital (a university-affiliated hospital providing regular blood transfusion, iron-chelation therapies and follow-up care for thalassemic patients in Tehran), or had undergone HSCT in the Hematology, Oncology and Stem Cell Transplant Research Center (HORCSCT) of Shariati Hospital (a tertiary care referral hospital in Tehran). All patients had diagnosis of thalassemia confirmed by hemoglobin pattern analyses and genotype studies. In order to be able to evaluate gonadal function, all the patients were required to have reached the age of 16 years at the time of evaluation.

Patients eligible for analysis in the post-HSCT group were those who had a complete hematological reconstitution and had survived for at least 1 year after transplantation. These patients had been prepared for transplantation with a myeloablative conditioning regimen including cyclophosphamide and busulfan. Regarding GvHD prophylaxis, cyclosporine (1.5 mg/kg daily, IV, on day ^− 2^, and then 3 mg/kg on day + 7) combined with methotrexate (10 mg/m2 on days + 1 and 6 mg/m2 on days + 3, + 6, and + 11) was administered. Cyclosporine was continued orally for at least 6 months after HSCT and discontinued in the absence of GvHD. No radiotherapy was given to any of these patients.

Ethical board of Tehran university of medical sciences approved the study and written informed consent was obtained from patients after a full explanation of the study.

### Fertility indicators assessments

Pubertal status was evaluated clinically according to clearly defined methods described by Tanner and Whitehouse and by scrotal ultrasound [11]. Testicular size < 4 mL (long axis of ≤2.5 cm) was considered stage I (prepubertal genitalia), and size > 25 mL (≥5 cm in length) was considered adult genitalia. Delayed puberty was defined as the absence of secondary sexual characteristics with a testicular volume of < 4 mL at the age of 14 years [12, 13].

By scrotal ultrasound, transverse and longitudinal images of each testis were obtained, and length, width and height measurements were made using electronic calipers. Testicular volume was calculated using the empirical formula of Lambert, length × width × height × 0.71 [14].

Blood samples were collected from patients for evaluation of their basal LH, FSH, and Testosterone (Ts) by immunoassay method. We defined hypogonadism according to the Endocrine Society definition for hypogonadism in men as a clinical entity that results from failure of the testes to produce adequate levels of testosterone and a normal number of sperms due to a disruption of the hypothalamic-pituitary-gonadal axis. We also categorized hypogonadism as primary or secondary. Primary hypogonadism was defined by low T and elevated gonadotropin levels (LH and FSH) [15, 16]. Hypogonadotropic hypogonadism was defined as LH and FSH levels< 2 IU/l, a Ts concentration < 3 ng/ml [17].

Semen specimens were collected after an abstinence interval of 3–4 days and were analyzed according to the World Health Organization guidelines. The following criteria was used to define the semen quality [18–20]:

The volume of ejaculate less than 1.5 mL was considered low. Azoospermia was defined as the absence of spermatozoa in patient’s ejaculate. Oligospermia was defined as a total number of spermatozoa below the lower reference limit (5th percentile, 15 million/mL).

Total motile sperm count (class A + B + C) fewer than 40% was considered abnormal. The percent of sperms with progressive motility (class A + B) less than 32% was considered abnormal. The percent of sperms with normal morphology less than 4% was considered abnormal.

Iron overload was assessed by measuring serum ferritin level, T2* MRI of heart and liver.

### Statistical analyses

Descriptive statistics such as frequency table, mean, standard deviation were used for analysis. Homogeneity between groups was assessed using Chi-square test for qualitative variables and Mann-Whitney U and Student t-test for continuous variables. A two-sided *P*-value of 0.05 or lower was considered to be statistically significant. Analyses were done with STATA version 11.2.

## Results

### Clinical characteristics

A total of 95 male patients were enrolled in this study, of whom 52 were transfusion-dependent and 43 had undergone allogeneic HSCT. The mean age of the patients in the transfusion-dependent group was 23.64 ± 6.11 years, and the mean age of the patients undergone allogeneic HSCT was 27.31 ± 4.34 years. The patients’ age range was between 16 to 41 years. The data on chronological age of the patientsand other clinical characteristics at the time of study are outlined in Table 1.

**Table 1:** Clinical characteristics of the patients involved in this study

			Transfusion-dependent patients	Patients undergoing HSCT	*p*-value
Number			52	43	
Age, years (mean ± SD)			27.31 ± 4.34	23.64 ± 6.11	0.84
Tanner Staging n (%)	P1		1 (1.9%)	1 (2.3%)	0.40
	P2		8 (15.4%)	9 (20.9%)	
	P3		18 (34.6%)	10 (23.3%)	
	P4		20 (38.5%)	8 (18.6%)	
	P5		5 (9.6%)	6 (14%)	
	missing		–	9 (20.9%)	
Right testis (mean ± SD)			11.10 ± 5.24	11.84 ± 9.22	0.005
Left testis (mean ± SD)			11.80 ± 5.77	11.06 ± 8.27	0.6
FSH (mean ± SD)			3.44 ± 2.12	8.17 ± 10.41	0.003
LH (mean ± SD)			4.49 ± 2.76	6.33 ± 3.82	0.22
Testosterone (mean ± SD)			4.61 ± 3.15	4.04 ± 2.57	0.1
TSH (mean ± SD)			3.67 ± 2.38	3.64 ± 2.64	0.7
T4 (mean ± SD)			7.59 ± 1.91	16.82 ± 3.67	0.003
Hypogonadism, n (%)			16 (30.8%)	15 (36.6%)	0.35
			10 (19.2%)	6 (14.6%)	0.38
Semen	Volume	Normal	38	9	0.00
		Low	9	23	
		Dry ejaculate	5	11	
	Viscosity	High	2	2	0.21
		Normal	44	29	
		Low	1	1	
		Dry ejaculate	5	11	
	Agglutination	Few	1	1	0.21
		Rare	1	1	
		None	45	30	
		Dry ejaculate	5	11	
Sperm	Concentration	Normal	31	16	0.017
		Oligospermia	9	5	
		Azoospermia	12	22	
	Total motility	Normal	31	16	0.017
		< 40%	9	5	
		Azoospermia	12	22	
	Progressive motility	Normal	26	14	0.017
		< 32%	14	7	
		Azoospermia	12	22	
	Shape	Normal	32	19	0.005
		< 4%	8	1	
		Azoospermia	12	22	

### Pubertal development

Tanner stage 4–5 was observed in 39 patients (41%), of whom 14 patients were in the HSCT group and 25 patients in the transfusion-dependent group. Among these males with adult genitalia, only 3 patients (7.6%) had hypogonadism and 2 patients (5.1%) had azoospermia.

Tanner stage 4–5 in the physical examination could be indicative of less possibility of hypogonadism and azoospermia compared to other stages (*p* < 0.001).

### Hormonal data

Hypogonadism was observed in 31 patients (32.6%), of whom 15 patients (36.6%) were in the HSCT group and 16 patients (30.8%) in the transfusion-dependent group. However, the prevalence of hypogonadism was not significantly different between the two groups (*P* = 0.35).

Most of them had hypogonadotropic hypogonadism, and only 4 patients from the HSCT group had hypergonadotropic hypogonadism.

The mean levels of LH, FSH and Ts were 5.30 IU/l, 5.53 IU/l, and 4.36 ng/ml, respectively. No significant differences were observed in the levels of LH and Ts between the two groups, while the difference between FSH mean level in TDTM patients was significantly lower than post transplant patients (3.44 IU/l ± 2.12 IU/l vs 8.17 IU/l ± 10.41 IU/l, *P* = 0.003). The mean LH and Ts level in those diagnosed with hypogonadism and those without it was 4.07 IU/l ± 4.63 IU/l and 1.30 ng/ml ±1.67 ng/ml versus 5.91 IU/l ± 2.35 IU/l and 5.89 ng/ml ±2.06 ng/ml, respectively.

Patients with hypogonadism had significantly lower sperm concentration (*p* < 0.001) and lower percentage of progressively motile sperms (*p* = 0.01).

### Semen analysis

According to the WHO reference [21], 16 patients (16.8%) had dry ejaculates. The prevalence of dry ejaculation was significantly higher in the HSCT group (11 cases, 25.6%) than in the transfusion-dependent group (5 cases, 9.6%; P  =  0.00).

Among all patients, 31 patients (32.63%) had unacceptable ejaculate volume (< 1.5 mL). The average volume of ejaculate in all patients was 1.55 mL.

Among those who were able to prepare a semen sample, normal ejaculate viscosity was seen in 92.4% and all of these patients had normal semen pH (7 to 8). Agglutination and prolonged liquefaction time (> 30 min) in ejaculate samples were seen in only 5 and 1.6% of patients, respectively.

Interestingly, azospermia, oligospermia, asthenospermia, teratospermia and even ejaculation with dry and low volume were all significantly more frequent in the HSCT group compared to transfusion-dependent group.

Significant differences were observed in the level of LH, FSH, and Testosterone among patients with azospermia between the two groups (Table 2).

**Table 2:** Evaluation of testicular function in azoospermia patients

Parameter	Azoospermia patients		*p*-value
	Transfusion-dependent group	HSCT group	
Mean LH (IU/L)	2.03	7.17	0.029
Mean FSH (IU/L)	1.7	14.65	0.002
Mean Testosterone (ng/ml)	2.59	5.82	0.031

### Testicular ultrasound

The mean volume of right testis was 11.39 mL and the mean volume of left testis was 11.51 mL and the mean size of testes was not different between the two mentioned groups of patients. Patients with lower testicular volume had significantly lower ejaculation volume, lower sperm concentration, lower percentage of motile and progressively motile sperm count as well as lower percentage of normal morphologic sperm. The frequency of hypogonadism was significantly higher in patients with lower testicular volume.

### Subgroup analysis

The fertility characteristics of thalassemic patients undergone HSCT based on their age at the time of transplantation (< 13 years or ≥ 13 years) was analysed. This age classification was performed according to data available in our literature review [22, 23]. In the post-HSCT group, patients’ age at the time of transplantation did not significantly impact their hormonal status and sperm parameters.

Chronic GVHD occurred in 14 (40%) of patients (limited/mild in 8 patients, limited/moderate in 3 patients, extensive/moderate in 2 patients, and extensive/severe in 1 patient). None of the patients had any genital GVHD. As shown in Table 3, no significant difference was observed between any degree of chronic GVHD and hypogonadism (*P* = 0.853). In addition, there was no significant difference between any degree of chronic GVHD and semen indicators.

**Table 3:** Characteristics of HSCT in thalassemia patients with offspring undergoing allogeneic HSCT

Patient	Age at transplant	Date of transplant	Sex	Donor Sex	Donor Type	Source of Stem cells	aGVHD	cGVHD	Conditioning
1	8	1991	Male	Male	HLA-identical sibling	BM	–	Mild Limited	Cyclophosphamide/busulfan
2	19	2008	Male	Male	HLA-identical sibling	Mesenchymal + PB	–	Moderate Extensive	Fludarabine/busulfan ATG
3	18	2009	Male	Male	HLA-identical sibling	Mesenchymal + PB	Grade 1	–	Fludarabine/busulfan ATG
4	26	2005	Male	Male	HLA-identical sibling	BM	–	Mild Limited	Fludarabine/busulfan
5	22	2008	Male	Male	HLA-identical sibling	Mesenchymal + PB	–	–	Fludarabine/busulfan ATG

In the transfusion-dependent group, patients’ ferritin level and T2 * MRI of the liver had no significant effect on their hormonal status and sperm parameters. However, in patients with more iron overload in the heart T2* MRI, sperm parameters were significantly lower and also hypogonadism was more frequent.

## Discussion

Allogeneic HSCT currently is the only definitive treatment for Beta thalassemia and the numbers of long-term survivors following HSCT have been noticeably increasing in recent years [24, 25]. These young patients desire to have children of their own [6–8]. Nonetheless, secondary complications in patients who underwent HSCT, are expected due to both primary disease and HSCT process [26, 27]. Cytotoxic agents used in the preparative regimen, cytokines release and infections during transplantation, post-transplant immunosuppression period, nutritional disorders, and psychological factors contribute to subfertility and hypogonadism [28–31]. In this study we compared the status of different indicators of fertility in thalassemia patients who had gone under HSCT with thalassemia patients who had continued the conventional treatment.

Primary hypogonadism due to iron overload is a common complication in TDMT patients and has been reported in approximately 50% of cases [32]. In a study by Safarinejad et al. the prevalence of hypogonadotropic hypogonadism was 76.2% and almost half of the male thalassemia patients had failure of pubertal development [33]. However, in thalassaemic patients after transplantation, the information is scant. Foregoing studies have shown a high proportion of primary gonadal dysfunction in patients with thalassemia disease after HSCT, of approximately 50–100% [34–37]. De Sanctis et al. reported that 40% of the evaluated patients with beta-thalassaemia major who had received a bone marrow transplantation during childhood or the peripubertal period, entered puberty normally, although most of them showed clinical and hormonal evidence of gonadal dysfunction [38]. In our study, hypogonadism was encountered in 32.63% of our patients but its frequency was not significantly different comparing the post-transplant patients with the TDMT patients (36.59% vs. 30.77%). It is notable that about 80% of post-transplant patients with hypogonadism actually had hypergonadotropic hypogonadism, while most of the TDMT patients had hyogonadotropic hypogonadism.

It has been proven that in males the germinal epithelium is more susceptible to cytotoxic agents than the Leydig cells [39]. The precise mechanism of the semen problem in beta-thalassemia patients seems to be very complex and relates to an unknown disorder of iron metabolism in patients [40]. Some researchers have found that more than half of men with thalassemia patients treated with blood transfusion and chelation are affected by abnormal sperm quality and oligospermia due to iron overload [41]. A study on the recovery of spermatogenesis after HSCT reported that spermatozoa were found in sperm fluid analyses in 27% of males [42]. Rovó et al. reported complete azoospermia in 69% of the evaluated male survivors after allogeneic HSCT. The major risk factors for sperm production after HSCT were total body irradiation and the most relevant adverse factor for sperm recovery was ongoing GVHD [43]. In our study, 64.21% of patients were not azospermic and interestingly, defects in sperm count, motility and shape and even imperfections in semen volume were all significantly more frequent in the post-transplant patients compared to TDMT group. In this study, we could not find any difference in hypogonadism between transplanted patients with cGVHD compared with those without cGVHD.

In the study conducted by Aldemir et al., they pointed out that HSCT age was a major important factor for preventing hypogonadism and early transplantation could decrease the risk of gonadal failure [23]. In a multicenter European study, comparing the age groups they reported that suspected infertility was less frequent in males who received HSCT treatment at the age of 13 or later than in those in whom treatment started at the ages of 1–12 [44]. However, in our study, patients’ age at transplantation did not significantly impact their hormonal status and sperm parameters.

Our study is unique in that we evaluated the fertility potential of thalassemic patients after HSCT in comparison with those who had continued their treatment with blood transfusion and iron chelators. On the other hand, using a non-TBI conditioning regimen, the impact of only chemotherapy regimens is evaluated. To our knowledge, there have never been reports of such a comparison. However, the present study has several limitations. The study design was cross-sectional. We studied a relatively small numbers of patients, especially in subgroups, which may affect the power of the study. Finally, we did not take into account the further laboratory tests in the patients with a suspicion of gonadal insufficiency.

## Conclusion

Although allogeneic HSCT is recognized as the only definitive treatment of TM, it seems to harm gonadal tissue and other aspects involved in fertility of male TM patients. It is imperative to conduct more research for recognition and overcoming the harms of HSCT on fertility indicators and improving the quality of life for HSCT survivors.

## Data Availability

The supporting data is available.

## Abbreviations

GVHD: Graft versus host disease
HSCT: Hematopoietic stem cell transplantation
TDMT: Transfusion-dependent major thalassemia
TM: Thalassemia major
